# The Brain Disorders Debate, Chekhov, and Mental Health Humanities

**DOI:** 10.1007/s10912-023-09786-1

**Published:** 2023-03-24

**Authors:** Jussi Valtonen, Bradley Lewis

**Affiliations:** 1 Department of Psychology and Logopedics, Faculty of Medicine, University of Helsinki, P.O. Box 21, Helsinki, 00014 Finland; 2 Theatre Academy, University of the Arts Helsinki, Helsinki, Finland; 3 Gallatin School of Individualized Study, New York University, New York, NY USA

**Keywords:** Mental health humanities, Chekhov, Neuroimaging, Narrative, Mad studies

## Abstract

The contemporary brain disorders debate echoes a century-long conflict between two different approaches to mental suffering: one that relies on natural sciences and another drawing from the arts and humanities. We review contemporary neuroimaging studies and find that neither side has won. The study of mental differences needs *both* the sciences and the arts and humanities. To help develop an approach mindful of both, we turn to physician-writer Anton Chekhov’s story “A Nervous Breakdown.” We review the value of the arts and humanities as a coequal partner with natural sciences in the creation of a robust mental health humanities.

## Introduction

In 2015, respected researchers Thomas Insel and Bruce Cuthbert coauthored an opinion piece in *Science* titled “Brain Disorders? Precisely.” The piece argued that, despite an avalanche of criticism, brain research and biological markers of mental disorders can be salvaged if research projects shift from current diagnostic categories to more rigorous natural science methods of measurable cognitive and neuroscience variables. Insel and Cuthbert’s optimistic faith in natural science, however, has not been supported by subsequent natural science research. The last few years have produced a wealth of findings pointing in exactly the opposite direction, showing that natural science approaches to mental health remain limited in their reach and that their results, despite remarkable financial and intellectual investments, have been largely disappointing. As a result, in 2019, a group of researchers countered Insel and Cuthbert with an article in *Behavioral and Brain Sciences* titled “Brain Disorders? Not Really…,” arguing that it is highly unlikely that mental illnesses can ever be conclusively explained in purely neurobiological terms (Borsboom, Cramer, and Kalis 2019, 1).

By this point, their conclusion has been reached by many others. “Neurobiology-based interventions for mental diseases and searches for useful biomarkers of treatment response have largely failed,” the prominent biomedical data scientist John Ioannidis (2019, 23) summarized. An editorial in the journal *Nature* noted that it seems that the “more scientists look for biomarkers for specific mental disorders, the harder the task becomes” (Across the Divide 2013, 398). This is not surprising, according to Ioannidis (2019, 23): “If mental health problems are mostly not brain disorders, the dearth of useful neuroscience-derived biomarkers is only to be expected.” Ioannidis argues that instead of looking harder, we should widen our perspective: “Instead of thinking of mental disease as a narrow problem of brain tissue, brain cells, and brain molecules, we may need to think of it as an evolving, ever-changing challenge for society at large” (Ioannidis 2019, 24). And Insel himself, in his latest book, *Healing*, is pulling back from brain research as the focal point toward the needs of the mental health system. His new hope for improving mental health involves turning our focus from brain science to “people, places, and purpose” (Insel 2022, xix). The road to recovery, Insel now believes, “to these three Ps” and “to a full and meaningful life” requires “something more” than medical care based on neuroscience and genetics (Insel 2022, xix).

Those familiar with the history and philosophy of psychology, psychiatry, and mental health studies will recognize that the brain disorders debate is not an isolated event. It is more like a contemporary outbreak of a conflict that has run through mental health studies at least since the late nineteenth century (Fulford, Thornton, and Graham 2006; Burston and Frie 2006; Walsh, Teo, and Baydala 2014). This tension, known in the literature as the *Methodenstreit* (German for “methodological debate”), centers on the question, What is the best way to study human beings? Should psychology, psychiatry, and mental health studies adopt the methods of the natural sciences (biology, physics, chemistry, quantitative social science, etc.), which focus on causal explanation, or should these fields adopt methods from the human sciences (literature, philosophy, history, the arts, etc.), which focus on interpretive understanding? Although the question is easy to ask, finding consensus on an answer has proven to be impossible for over 100 years of mental health study. The result has been conflicted and fragmented research and practice domains that emphasize either natural sciences approaches (neurological, genetic, behavioral, cognitive, etc.) or human sciences approaches (phenomenological, psychoanalytic, narrative, hermeneutic, feminist, postcolonial, etc.).

We argue that the wealth of contemporary findings on the limits of brain science provides an ideal moment for a ceasefire in the century-long *Methodenstreit*. Rather than continue this conflict and perpetuate a fragmented field of mental health studies, now is the time to add the arts and humanities as a full and respected partner in the ongoing work of understanding and making improvements to our mental lives. This does not mean that either side—the arts and humanities, on the one hand, or the natural sciences, on the other—has won the conflict; it simply means moving from conflict to collaboration and cooperation. The lesson of over 100 years of conflict is not to declare a winner but to accept that both sides have legitimate contributions. Now is the time to embrace both sides of this conflict instead of choosing between them and to build a democratic scaffold where both the natural sciences and the arts and humanities are included in the study of mental health.

Our argument for including the arts and humanities in mental health studies has three parts. We first review recent findings from one subfield of the contemporary brain disorders debate—functional neuroimaging—illustrating the current limits of some of the most promising natural sciences approaches to mental difference. Our goal in this review is not to invalidate ongoing brain research but, rather, to show that we have learned something significant from the findings to date—namely, that the brain disorders approach is limited on its own and future yields will be slow. The research is invaluable in showing us what can be learned using brain-research methods and what can be gained by incorporating additional approaches. Our next step is to turn to the work of Anton Chekhov (1860–1904), particularly his short story “A Nervous Breakdown,” for additional possibilities. Chekhov stands out, both through his art and his dual engagement with medicine and literature, as a pluralistic advocate of both science and literature. Chekhov, who worked in a time before the methodological conflict had hardened, used an approach to mental differences in his stories that values the sciences but also exemplifies the possibilities of the arts and humanities. In this way, Chekhov can be a guide for mental health research and practice at a time when biological answers are turning out to be much more complicated than early proponents of neuroimaging had hoped. Finally, generalizing from Chekhov’s contribution, we develop an arts-and-humanities approach to mental health and mental difference through the articulation of interdisciplinary mental health humanities as a significant contributor to future mental health research, education, and practice. In this last section, we review the value of the arts and humanities more broadly and then focus on how to bring these domains to mental health.

## Brain disorders debate: research findings

When modern functional neuroimaging techniques became available, many researchers were optimistic that methods such as positron emission tomography (PET) and functional magnetic resonance imaging (fMRI) would soon facilitate new scientific breakthroughs in the understanding of mental illness. This possibility was greeted with enthusiasm, in part because it was already becoming clear that investigations of both neurotransmitter function (Healy 2015; Lacasse and Leo 2015; Moncrieff et al. 2022) and candidate-gene associations (Border et al. 2019; Charney 2022) had come to a dead end, not having resulted in any clinically useful findings despite massive research efforts. An article in *Nature* concluded in 2013 that “despite decades of work, the genetic, metabolic, and cellular signatures of almost all mental syndromes remain largely a mystery” (Adam 2013, 417). With the advent of neuroimaging, however, prominent psychiatric researchers shifted their attention from these prior lines of research to functional neuroimaging techniques. Insel and Quirion (2005, 2222–23) described the shift in perspective this way: “Ultimately, biomarkers for mental disorders may not be proteins or neurotransmitters but may emerge from neuroimaging ([fMRI], [SPECT], etc.). Logically, if these are disorders of brain systems, then the visualization of abnormal patterns of brain activity should detect the pathology of these illnesses.”

The hope was that neuroimaging studies would, once empirical evidence accumulated, identify aberrant brain-activation patterns in conditions such as depression, even if earlier biocentric research programs had not succeeded. Unfortunately, the hope for fundamental breakthroughs is still unfulfilled. Unlike when Insel and Quirion expressed their optimism about future studies, functional neuroimaging techniques have now been available for several decades—and yet the pathology of mental disorders remains elusive.

In the example of depression—a key aspect of mental health studies as a whole—an important benchmark is a series of meta-analyses evaluating all the empirical evidence that has been collected over the past two decades regarding aberrant brain-activation patterns in major depression. While several such patterns have been reported, the findings have been highly inconsistent across studies (Fitzgerald et al. 2008; Diener et al. 2012; Graham et al. 2013; Lai 2014; Palmer et al. 2014), casting doubt on their validity. Therefore, in a series of important meta-analyses, Müller et al. (2017) sought to identify what had been established in all neuroimaging studies to date. To that end, they pooled together all studies published between 1997 and 2015 that used PET or fMRI to investigate depression-related changes in brain activity in cognitive and emotional processing. Disappointingly, this analysis found simply that these studies had failed to establish any reliably replicable patterns across experiments: according to Müller et al. (2017, 4), all functional neuroimaging experiments investigating unipolar depression conducted to date, when considered together, “did not reveal any convergence” across findings. According to the authors, this failure may be related to differences in experimental design, misguided statistical procedures, heterogeneity among patients, or all of the above.

Perhaps even more sobering is that this disappointing inconsistency across studies is not limited to studies of unipolar depression, as the authors point out. In part, the difficulty in finding consistently identifiable brain activation patterns across experiments is related to much broader concerns. That is, there is increasing and serious concern over the replicability and reproducibility in neuroimaging research in general (Button et al. 2013; Barch and Yarkoni 2013; Szucs and Ioannidis 2017; Poldrack et al. 2017; Chambers 2019). This is also what Müller et al. (2017) conclude from the past two decades of neuroimaging studies on depression: that researchers should focus on improving the reproducibility of results in future studies. The point was further accentuated by a recent analysis of brain-wide association studies published in *Nature* in 2022, analyzing the brain-behavior correspondences of more than 50,000 brain-research participants (Marek et al. 2022). The authors concluded that most published brain-wide association studies that have sought to draw a link between patterns from brain imaging and behavioral features, such as mental differences, have not been reliable. The neuroimaging community is thus currently engaged in an ongoing debate about the best new practices that should be adopted to ensure more reproducible research results in the future (Munafò et al. 2017; Müller et al. 2018; McIntosh and Chambers 2020).

To be clear, these sobering findings do not mean that there is anything wrong with brain research. On the contrary: this is how scientific progress occurs, through the rigorous testing and falsification of empirical claims. The situation does stand in stark contrast, however, with the early research optimism that neuroimaging inspired when the methods first became available. More than two decades of PET and fMRI studies have neither discovered the true pathology of depression nor redefined psychiatry as “a clinical neuroscience discipline,” contrary to what Insel and Quirion (2005, 2224) envisioned. Instead, we are left with no reliably identified brain activation patterns for any mental health conditions, and the situation is still the one described in an editorial in *Nature* nearly a decade ago: “Genetics and neuroimaging studies would, all involved hoped, reveal biological signatures unique to each disorder, which could be used to provide consistent and reliable diagnoses. Instead, it seems the opposite is true. The more scientists look for biomarkers for specific mental disorders, the harder the task becomes” ("Across the Divide" 2013, 398). Neuroimaging has indeed fundamentally “reformula[ted] our notions” (Insel and Quirion 2005, 2223) but, ironically, in a way opposite to what Insel and Quirion envisaged 18 years ago: the research is showing primarily that strictly biological answers are not to be quickly anticipated from this line of research and that we, therefore, have a pressing need for additional methodological approaches.

Even more importantly, perhaps, it is not entirely clear how useful brain activation patterns would be for explaining mental distress were we able to find them, even if they were robust and perfectly replicable. Functional neuroimaging methods are best suited for *localizing* identified mental functions to particular areas of the brain—not for uncovering how humans process, interpret, and make meaning of their experiences (Coltheart 2010). The common belief that neuroimaging can *explain* human behavior is, by and large, a misunderstanding of how these tools work (Beck 2010). Therefore, even if we could use neuroimaging tools successfully to localize some aspects of mental distress to networks in the brain, they would not do much in themselves to explain them, let alone render any other dimensions of mental suffering and mental difference irrelevant.

Functional neuroimaging, investigations of neurotransmitter function, and genetic research have taught us a great deal about the brain and neurophysiology, and they may yet discover unanticipated truths about mental differences that get labeled “mental illness.” However, regardless of whether or when such new truths may be uncovered, current research findings from neuroscience strongly encourage us also to consider other dimensions of mental life and other methods of understanding ourselves. While it may be a fairly uncontroversial view that purely reductivist neurogenetic approaches will not take us very far in understanding mental distress, the risk of remaining stuck in overly narrow frames persists as long as the human- versus natural-science sides of the century-long *Methodenstreit* remain isolated and in conflict. To illustrate what we mean, we turn to a short story by Anton Chekhov, which we believe can provide a more expansive frame for thinking.

## Chekhov’s perspective

Anton Chekhov, well known for his stories and dramas, was also a physician, and he took both worlds—literature and medical science—seriously. His dual positions in art and science come together in his writing to create a hybrid perspective beyond what we usually see in either domain (Coulehan 2003; Lewis 2011; Fisher 2017). Chekhov did not write out this perspective in an academic form, but it is possible to understand his approach to mental difference from his plays and stories. The short story we focus on here is about a law student, Vasilyev, who has a “nervous breakdown” after an encounter with the world of paid sex.

In retelling the story, we have not reduced it to a case history as one might see in a diagnostic training manual; instead, we have retained some of the literary elements of Chekhov’s writings. Chekhov’s story reveals that these literary elements cannot be abstracted or summarized to retain the same meaning. Developing a detailed understanding of the particularity of the human experience is at the heart of many approaches in the arts and humanities, and this goal is increasingly lost with increasing abstraction. While it is often helpful to discuss literary works at the level of thematic interpretations or plot summaries, the human singularity of the events and their impact on the protagonist, conveyed in narrative form, are critical here for the larger point we wish to make.

Chekhov’s story, widely and aptly titled “A Nervous Breakdown,” begins late one evening with three young men heading out for a night on the town (Chekhov 2014, 183). The air smells of freshly fallen first snow; the whole city looks soft, white, and young. The medical student Mayer and the art student Rybnikov have convinced their friend, the law student Vasilyev, to go to a part of the city known as S. Street. Despite his agreement, Vasilyev, who knows the street’s reputation, is reluctant and has resisted going in the past.

“No philosophizing, please,” says the medical student when they’re at a restaurant having drinks. “Vodka is given us to be drunk, sturgeon to be eaten, women to be with, and snow to be walked upon. Live at least one night like a human being!”

“Don’t worry … I’m not trying to get out of it,” says Vasilyev.

Watching his friends in the restaurant, Vasilyev feels a combination of envy and admiration. The other two look strong, healthy, and cheerful—unlike Vasilyev, who watches “over every step, his every word,” is “distrustful” and “cautious,” and “elevates every trifle to the level of a problem.” Vasilyev longs for “at least one night to live like his friends, to let himself go, to free himself from his own control” (184–85).

He decides that if he is taken to the women, he will go.

The sex trade in the S. Street brothels turns out to be straightforward and tawdry. Vasilyev is shocked at the banality of prostitution and baffled by the women working in the sex industry. Contrary to what Vasilyev has imagined, the women seem neither helpless victims nor fallen angels. When he tries to initiate a conversation with the women, he fails to make any sense of how they have ended up on S. Street or how they feel about their lives. Vasilyev finds the experience profoundly unsettling. He feels infuriated by his friends’ behavior and guilty about his own role in the exploitation of the women.

After returning home, Vasilyev ruminates over how to help the women. The problem seems enormous. Brothels don’t exist only on S. Street. They are all over the world. Moreover, the arrangement is highly dependent on demand as well; to make a difference, he would probably need to find a way to intervene with the clients too. The more he broods over the scale and social complexity of the situation, the more aggravated he becomes. Even saving one single woman from exploitative and often forced sex work seems impossible. Eventually, he is so beside himself with frustration that he can no longer eat or sleep. He becomes deeply distraught with inexplicable and crushing terror, unbearable mental anguish, despair, and compelling thoughts of self-harm and suicide. He pleads with his friends: “Take me wherever you want, do whatever you can, but for God’s sake hurry, save me or I’ll kill myself!” (200).

The medical student knows a psychiatrist, “a plump fair-haired doctor” with “a huge practice,” and they take Vasilyev right away. The psychiatrist greets them “politely, respectfully, coldly and smiled with only one cheek.” He proceeds to ask questions about Vasilyev’s father—“Had he been ill?” Was he “a binge drinker?” Was he “cruel or strange?”—and then to ask similar questions about Vasilyev’s grandfather, his mother, his sisters, and his brothers. The psychiatrist takes careful notes as he goes and eventually finds out that Vasilyev’s mother was a singer and had occasionally acted in the theater. The psychiatrist is particularly fascinated with this information: “He suddenly came to life, ‘Sorry but do you recall if the theater was a passion for your mother?’” (200).

Vasilyev becomes bored with this line of questioning and eventually exasperated: “As far as I can make out from your questions, doctor … you want to know if my illness is hereditary or not. It is not hereditary” (201).

The psychiatrist moves on to ask about Vasilyev’s childhood—whether he had any “secret vices, injuries to the head, odd behavior, or exceptional properties”—and then about his current schoolwork. He learns “that Vasilyev had already finished the natural sciences and was now a law student.” With this last information, “the doctor grew pensive,” and Vasilyev’s friends try to help by adding that Vasilyev is a particularly good student: “Last year he wrote an excellent paper.” But the psychiatrist stops them abruptly, “Please don’t interrupt me, you’re keeping me from concentrating.” Vasilyev tries to explain that what is worrying him most is prostitution, the plight of the women he met, and the whole question of whether “prostitution [is] an evil or not” (201). The psychiatrist shrugs it off as an uninteresting concern. Of course prostitution is an evil, but it’s just the way things are.

Vasilyev can’t understand this response and starts pacing back and forth, “I’m praised to the skies because I wrote a thesis that will be thrown away and forgotten in three years. But because I’m unable to speak about fallen women as matter-of-factly as I might about these chairs, I’m being treated medically. They say I’m crazy, they feel pity for me!” (202). He finds himself all alone and completely alienated, and by now it is clear that even the psychiatrist is hopelessly out of tune with his concerns. Vasilyev “burst into tears and fell into the armchair” (202). His friends look in confusion to the psychiatrist for help. But the doctor is not impressed:

He wore an expression as if he perfectly understood both the tears and the despair, as if he considered himself an expert in such matters. He approached Vasilyev and without a word gave him some drops, and then, when the patient had calmed, he disrobed him and began to test the sensitivity of his skin, his knee reflexes, and so on. (202)

Surprisingly, although Vasilyev feels embarrassed by the whole procedure, he also does start to “feel a little better.” (202) He pockets the prescriptions for bromide and morphine, says goodbye to his friends, and heads back toward the university. The crisis has passed, but the story makes clear that the crux of Vasilyev’s situation, like the problem of prostitution, has not been resolved or even meaningfully understood. Vasilyev has gotten little help in the end. Chekhov does not show us whether his suffering will become chronic, but the reader feels this is a real possibility. If Vasilyev does eventually find a way to navigate the difficulties of the world after the story ends, Chekhov makes it clear this does not happen because of the psychiatrist’s clinical-biological expertise.

From this presentation, we can see that Chekhov’s story “A Nervous Breakdown” is both a depiction of a student perplexed and distraught by the social oppressions of exploitative and often forced sex work and an incisive portrayal of the standards of care used by the psychiatrist who tries to help. Both situations are tragic, and they become doubly tragic when combined in the clinical encounter. Vasilyev is clearly suffering and desperate for help when he pleads with his friends to take him to a professional. His friends seem to care for him and to be well-meaning, but they do not understand Vasilyev’s concerns. Vasilyev’s cry for professional help is a cry for someone trained in that very task—to be able to understand mental states that not everyone shares. The medical student picks a seemingly competent psychiatrist who is well respected, teaches at the medical school, and has a large practice. But the psychiatrist only barrages Vasilyev with a series of scientifically predetermined and reductionist questions. He seems blind to Vasilyev’s experience or the possibility that Vasilyev’s concerns are valid, and he fails to take an interest in the very social and political issues behind Vasilyev’s distress and despair. Vasilyev now feels completely alone with his troubles as not even the professional has a clue to his state of mind. Not only that, but the psychiatrist also ends up using toxic drugs (bromide and morphine) to “calm” Vasilyev with no mention of potential side effects and risks of taking these medications in the longer term.

It is important to see that Chekhov does not portray the psychiatrist as a “bad apple” but rather as a representative of his field. The psychiatrist follows clinical practice protocols self-importantly, the very ones his colleagues have plumed themselves with as well. He systematically goes through a long list of questions intended to ensure a careful anamnesis and an objective diagnosis, and official guidelines and treatment protocols are undoubtedly followed. Literary scholar Cathy Popkin (2006, 113) points out that Chekhov is writing in “response to prevailing psychiatric practice and as a kind of corrective to the genre—the poetics, as it were—of the psychiatric case history.” Comparing this with other stories Chekhov read in the newly minted psychiatric press at the time, Chekhov clearly pulls away from the neurological reductionism prevalent in Russian psychiatry in his time. Through his narrative describing how Vasilyev’s breakdown came about, according to Popkin, Chekhov attempts (among other things) to rectify psychiatric case histories, which typically failed to tell patients’ stories—to elucidate how they ended up where they were.

In a very similar way, Chekhov pulls away from the current biocentric paradigm of seeing mental distress primarily through the lens of the natural sciences. Although most (Chekhov’s psychiatrist and contemporary mental health professionals included) would surely agree that individual circumstance, history, and perspective are important for understanding and treating mental distress, Chekhov highlights how easily this becomes mere lip service. The particularity of each individual and their singular situation is abstracted away by definition in contemporary psychiatry. The Diagnostic and Statistical Manual of Mental Disorders, the DSM, strives for categories that are presumably “more objective” in themselves and that can later be translated into the even “more objective” language of neurobiology and genetics. But a well-identified problem in this approach is that it has created “epistemic blinders” that obscure other viewpoints, limiting our understanding of mental distress (Hyman 2010, 155). Chekhov’s fictitious psychiatrist is a timely depiction of such blinders, historical and contemporary, created by the pursuit of objective natural sciences knowledge that becomes detached from human meanings.

In this way, Chekhov’s story immediately opens to the very contemporary concerns we saw in the brain disorders debate: it depicts, in story form, not only the factors underlying a singular human episode of mental distress but also the consequences of mental healthcare that fails to move past the dominant biomedical and natural science model. Moreover, it suggests a range of ways forward for clinical research, education, and practice. At its most basic, moving past a narrow biomedical natural science model means opening to increased content, consistent with a biopsychosocial or an ecosystems perspective (Engel 1977; Kirmayer, Lemelson, and Cummings 2015). Chekhov demonstrates the need for such an expanded frame in “A Nervous Breakdown” by highlighting a range of sociological, psychological, and biological contributions to Vasilyev’s situation. But Chekhov goes further than Engel’s biopsychosocial model by bringing out political, spiritual, and aesthetic dimensions as well. And, as we will see, Chekhov goes further still by bringing in narrative and hermeneutic processes of meaning-making and the hermeneutic question of whether and how much to pathologize Vasilyev’s difficulties. The result is an approach to mental difference that includes an expanded range of variables beyond the biopsychosocial model along with an inclusion of the interpretive processes for understanding these factors and their interplay.

Starting with the social and political factors involved in Vasilyev’s difficulties, Chekhov highlights the importance of the young women working at the brothel. The women who trigger Vasiliyev’s concerns are ordinary human beings abandoned by a corrupted society. Neglected and forgotten, they are left to deeply dysfunctional social suffering stemming from the “environment, poor upbringing, poverty, and so on” (Chekhov 2014, 183). These large-scale social and political forces are compounded by Vasilyev’s immediate interpersonal world—his friends, who do not show concern for this kind of obvious social trauma and political oppression. The friends know that, medically, “every one of these women will die prematurely from tuberculosis or something else” and that morally they will die even sooner (195). Yet they simply feel the way most people feel—that this is the way things are, that it is no real concern of theirs. Worse, they blithely participate in the abuse, “exploiting hunger, ignorance and stupidity” for a night’s entertainment and pleasure (183).

In addition to these social and political causes, another aspect of Vasilyev’s anguish is personal or psychological. Compared to “a normal person,” as Vasilyev uses the term, he is more sensitive to the plight of the women, and their social suffering throws him off balance (Chekhov 2014, 187). He is unable to put the conflict out of his mind, and he works diligently to find solutions for this kind of systemic abuse. Vasilyev finds the problem so complicated, however, that he is overwhelmed by the impossibility of doing anything meaningful to help. Vasilyev, the narrator tells us, has a special “talent for *humanity*. He possesses an ultrasensitive, exquisite sense of pain in general. … [He] is able to reflect in his soul another person’s pain” (197–99, emphasis in original). In addition, Vasilyev feels a deep compulsion to act on his sensitivity, to respond as if he were “the brother of a fallen woman” or “her father” or even “the fallen woman herself with painted cheeks” (196). Vasilyev strives “to resolve this problem immediately, no matter what," because he feels “that it was not somebody else’s problem, but his very own” (196).

What is important to see is that Chekhov does not simply pathologize or romanticize Vasilyev’s difference from the norm here. Compared to the norm, Vasilyev is more sensitive to social suffering, and he yearns for a world with more justice. The injustice that concerns him is not just a social problem; it is a political problem. It is a political problem that has become a personal problem. But unlike common uses of a biopsychosocial model, Chekhov does not see Vasilyev’s sensitivity and maladjustment to these political problems as psychopathology in any straightforward way. Political problems need people who are maladjusted to the norm, who are sensitive to the plight of the oppressed and subordinated, and who yearn for a better world. As Martin Luther King so memorably put it:

There are certain technical words in the vocabulary of every academic discipline which tend to become stereotypes and clichés. Psychologists have a word which is probably used more frequently than any other word in modern psychology. It is the word “maladjusted.” This word is the ringing cry of the new child psychology. Well, there are some things in our social system to which I am proud to be maladjusted and to which I suggest that we ought to be maladjusted.

I never intend to adjust myself to the viciousness of lynch-mobs. I never intend to become adjusted to the evils of segregation and discrimination. I never intend to adjust myself to the tragic inequalities of an economic system which takes necessities from the masses to give luxuries to the classes. I never intend to become adjusted to the madness of militarism and the self-defeating method of physical violence.

History still has a choice place for those who have the moral courage to be maladjusted. The salvation of the world lies in the hands of the maladjusted. (King 1997, 285–86)

From this perspective, Vasilyev’s psychological difference from the norm is not a pathology. It is a valuable maladjustment because only through maladjustment do we have hope of changing the deep political problems of human exploitation. We can, of course, insist on labeling it a pathology because of some of its consequences, but this is a hermeneutic choice that is not value-neutral. Contemporary psychiatry typically does not grapple with this choice or its implications, perhaps because the tools that would help in doing so are mostly in the fields of human sciences or perhaps because many in the field have wanted to believe psychiatry has been able to circumvent value-laden choices by embracing the natural sciences. Chekhov, by contrast, forces the reader to face this question, among others, head-on.

Vasilyev, also like Martin Luther King, approaches his maladjustment to political problems from a spiritual perspective or what Chekhov’s (2014, 198) narrator, moving beyond a biopsychosocial model, calls “spiritual agony.” Vasilyev imagines himself doing “missionary work” and feels “a searing love for those people who would heed his words and stand alongside him preaching” to end this terrible social suffering (Chekhov 2014, 198). From a spiritual perspective, Vasilyev’s difference—that is, his heightened sensitivity—can be seen as a gift rather than a pathology because it can become a trigger for social change that can take a spiritual or political form. It can also take an aesthetic form—as in Chekhov’s story “A Nervous Breakdown” or Martin Luther King’s eloquent and moving prose.

But this sensitivity can also be a deep challenge. None of this less pathological, more celebratory, dimension of Vasilyev’s mental difference means that his sensitivity and yearning are easy to live with and are not the source of considerable difficulties—even symptoms, suffering, and disability. Moreover, the consequences of such sensitivities profoundly depend on the outlet(s) the person finds—or fails to find—for acting on the social, political, ethical, spiritual, aesthetic, or other issues to which they are sensitive. Taking into consideration the various layers of personal and other variables that are at play does not mean, for example, ignoring the very real risk of self-harm in Vasilyev’s case. Chekhov’s anti-pathological, anti-sanist framings of the struggles of mental difference do not romantically erase the challenges of difference. But they do create meaning-making options beyond pathologizing models and help counterbalance the (currently predominant) impulse to situate sources of psychological distress removed from its political, spiritual, and aesthetic context (Lewis 2017).

Finally, biology also enters the picture because, eventually, Vasilyev’s conflict leads to a lack of sleep, difficulty with self-care, and a serious impact on Vasilyev’s body. Thus, biological variables clearly play a role in the form that Vasilyev’s distress takes. In addition, biology is also present in the limited benefit Vasilyev receives at the hands of a biologically reductionistic psychiatrist. The improvement may be a placebo, but it comes from a social interaction in which a biomedical intervention has been recommended. The story is open to the possibility that biological treatments could be helpful, at least over the short term—Vasilyev has taken these same medications before—but the story also shows the negative consequences of trying to push biological variables repetitively beyond their interpretive power.

We can see from this review that a biopsychosocial or ecosystems model is consistent with Chekhov’s approach, but it is also clear that an ecosystems model is not enough. Chekhov’s ability to go further than most biopsychosocial approaches to include variables of politics, spirituality, aesthetics, and nonpathological framings of mental difference shows that there is more going on here than even in an expanded ecosystems frame, let alone in a narrow brain disorders model. This is where we start to see the importance of Chekhov’s writing practice and his dual engagement with medicine and the interpretive arts of literature. Chekhov’s story adds psychological, social, political, aesthetic, and spiritual content, yes, but it also adds attention to the narrative and hermeneutic processes of choosing and ordering this content.

For Chekhov, the only way to organize the many elements that could be brought to bear on problems of this complexity is through narrative and interpretive choices. Natural sciences, as invaluable as they are in their own realm, cannot provide the tools needed for this. If we want to formulate answers to key clinical questions—such as How did this problem come to be?, What variables and factors should we use to make sense of it?, What models of meaning (bio-psychiatry, psychoanalysis, cognitive behavioral therapy, family, feminist, spiritual, creative-expressive, etc.), or combinations of models, should we use?, What should we do in response to the real suffering and risks?, and, most important, What does/do the person/persons most affected want?—we must use an interpretive process of narration (Lewis 2011). Not only that, if we are going to ask questions about how to prioritize our research methods and funding we must use interpretive processes. As Popkin puts it, “Giving form to pain (whether one’s own or someone else’s) is the only hope of connection and communication, but also of diagnosis and treatment. … [For this,] narration and representation are not alternatives to medical science but essential to it” (Popkin 2006, 118).

Popkin’s understanding of Chekhov, in an essay she titles “Re-stor(y)ing Health,” lines up nicely with a wealth of Chekhov research that is reaching exactly the same conclusions (Lewis 2011; Fernandes 2015; Fisher 2017). Chekhov’s frame for mental difference likely emerged from his personal experience of combining medicine and literature. It was through the constant movement back and forth between medicine and writing that Chekhov broke out of the standard frame of most medical thinking to include an arts-and-humanities frame for mental difference. He was able to use his dual position of doctor and writer in a unique way to produce diametrically opposed relationships to the role of interpretation.

In his occupation as a doctor, Chekhov’s task was to background narrative frames and to view his patients from a positivist model of objectivity. But this positivist stance was not Chekhov’s only position. As a writer, he worked from an opposite position that foregrounds narrative and hermeneutic frames. In other words, he inhabited a practice that highlights the impossibility of telling a story without a point of view. What is interesting is that Chekhov reached this understanding not only from literature but also from medical reform efforts he was exposed to in medical school. Indeed, the mantra he inherited from his training was exactly this: “Do not treat illness as if it were identical for everyone. … [T]reat the patient with all his individual peculiarities” (Kataev 2003, 94).

Chekhov adopts this theme from his medical school instructors working against the grain of clinical dogmatism in their time. Chekhov learns the motto of “rigorous individualization” of each case and “the uncompromising rejection of stereotypes in treatment” (Kataev 2003, 95). As Chekhov has one of his characters put it in “A Boring Story”:

My therapeutic colleagues … tell all their students to "individualize each specific case." One has only to take this advice to realize that the remedies recommended in textbooks as the best, and entirely suitable as a standard rule, are quite unsuitable in individual cases. The same applies to moral ailments. (Chekhov 1964, 92)

## Mental health humanities

Chekhov’s approach to mental difference opens mental health work beyond bioscience and beyond the biopsychosocial model to a wealth of interpretive arts-and-humanities perspectives. To build on Chekhov’s mental health humanities perspective, we put his work together with contemporary considerations of the humanities. Helen Small’s *The Value of the Humanities* summarizes five common arguments for the humanities (Small 2013; adapted from the conclusion):

1. They do a distinctive kind of work.
2. Their work is useful to society.
3. They contribute to individual and collective happiness.
4. They contribute to the maintenance and health of democracy.
5. They are good for their own sake.

These arguments distill a century of scholarship on the value of the humanities for society, and they can be used to help clarify the value of the humanities for mental health work as well. We emphasize, as does Small, that when we use the term *humanities* we are referring to *arts and humanities*. This paper is an obvious example. Chekhov’s story (art) and this article’s commentary (humanities) are both relevant to *mental health humanities.*

Small’s first argument is that the humanities bring a distinctive kind of *knowing* that cannot be achieved through other approaches. This specificity-of-knowing argument is bound up in the very definition of what the humanities do. Small works out a shorter and a longer version of the distinctive work of the humanities. The shorter version describes the humanities without reference to the sciences: “The humanities study the meaning-making practices of human culture, past and present, focusing on interpretation and critical evaluation, primarily in terms of the individual response and with an ineliminable element of subjectivity (Small 2013, 58).

Very much as we saw in Chekhov’s “A Nervous Breakdown,” the arts and humanities study the human condition, the lived experience of what it is to be human, and the larger context and history of human cultural practices. The arts and humanities do this through interpretation, aiming to understand what it is to be human in different settings and with a critical eye on the implications of what is being studied. There is a double subjectivity involved in this kind of humanities work—both the inescapable subjectivity of human life itself and the subjectivity of the scholar or artist of human life.

The longer version works out the distinction between this type of humanities work and the sciences:

In the main the humanities value qualitative above quantitative reasoning; they place greater faith in interpretative than in positivistic thinking; unlike the sciences and the scientific wing of the social sciences they do not have a dominant methodology, and many of their truth claims are not verifiable … ; they tend, accordingly, to distrust proceduralism and to value independence of thought. They are orientated as much toward historical analysis as toward synchronic structural analysis, and as much toward the medium of expression as towards its content (tending to see the form/content distinction as itself problematic). They attend to the role of the perceiver in ascertaining even the most philosophically secure of knowledge claims; and they have an interest, often they also take pleasure, in the specificity of the object of study and the specificity of the individual response (its content and its style) over and above the generalized or collective response. Not least, they respect the products of past human endeavors in culture, even when superseded. (Small 2013, 58)

In this longer version, like in Chekhov’s story, Small places the specificity and distinctness of the humanities in contrast with the objects and methods of the natural sciences. Similarly, the way Chekhov’s story understands Vasilyev is very different from that of the scientific psychiatrist in the story. There is also a clear echo between the distinctness of the humanities as they are practiced and the *Methodenstreit* discussed earlier. Most contemporary psy disciplines have prioritized the causal/natural-science side of the *Methodenstreit* (Teo 2017), but the understanding/humanities side of studying the human condition has not disappeared. Like Chekhov’s story, it has simply continued in the arts and humanities, segregated from the dominant psy disciplines. This means that the kinds of knowing that the arts and humanities engage with remain a resource that can be critically drawn on for a more multifaceted understanding of mental health.

The second value that Small articulates for the arts and humanities is their usefulness to society. Small focuses her chapter on Matthew Arnold’s *Culture and Anarchy* (2006; originally published in 1869). Arnold saw the arts and humanities as a treasure of “the best which has been thought and said” that is invaluable for the development of human culture, character, and civilization (Arnold 2006, 5). In the last 50 years, scholars have largely dismissed Arnold for his high cultural slide into elitism, racism, and snobbery. Small, however, cautiously sees opportunities to bring back “usefulness” and “instrumental value” to our understanding of the arts and humanities by arguing for a “modern ‘Arnoldian’ cultural contract that need not bring in its train his high cultural assumptions” (Small 2013, 60).

Narrative psychologist Mark Freeman provides a good example of this more modest cultural contract in his discussion of psychological humanities. According to Freeman, “human life has a literary structure” and therefore the value of the arts and humanities for psychology is that they are “crucial to understanding human lives” (Freeman 2020, 30). Freeman gives two observations near the surface of everyday experience to justify his claim. First, “novels, memoirs and autobiographies, and certain forms of poetry … [appear] to be the most ‘natural’ vehicle for exploring and understanding human lives.” Second “literature is often able to speak to the complexities of human lives with an urgency, an intensity, and an evocative power scarcely found in psychology” (Freeman 2020, 30). These observations point to a deep connection between the arts and humanities and what Freeman (2017, 25) calls “life itself.” From this perspective, life itself “is a narrative achievement, the stories we actually tell serving to extend and refine the storied movement of this very life” (Freeman 2017, 15). This use value of the arts and humanities is the very message Chekhov’s “A Nervous Breakdown” imparted to us over 100 years ago.

Small’s third argument is that the arts and humanities can bring happiness and well-being at the individual and collective levels. Small treads cautiously with this argument and calls it the “least trusted line of defense” (Small 2013, 6). But nonetheless, she explores three aspects of the happiness argument. The arts and humanities provide (1) a deeper understanding of happiness, (2) enriched character development, and (3) direct pleasure or hedonism. We, as authors, have an intuition on all three in relation to “A Nervous Breakdown.” We feel that reading Chekhov gives us an increased understanding of happiness, an enriched character, and a direct experience of aesthetic pleasure. Not everyone would feel this way, so it is hardly universal, but at least some people do. Small helps us work through these intuitions in greater detail.

Starting with the first argument for happiness, the arts and humanities may increase happiness by “deepening our *understanding* of what happiness consists in, how we may best hope to attain it, what the relation may be between the psychological happiness of the individual and the well-being of society, and how education may alter the quality as well as the range of pleasures available to the individual” (Small 2013, 176; italics added). The key word here is *understanding* because it links us back to the distinct role of the arts and humanities for an interpretive understanding of the human condition more generally. If the humanities provide a form of understanding we cannot get from the sciences, one of the key places where that understanding can be brought to bear is the question of human happiness. There is a complex and contentious empirical literature in positive psychology devoted not only to happiness but also to related terms such as *well-being*, *flourishing*, *eudaimonia*, and *mental health*. The argument for the humanities is that we can better sort out the meanings of these key aspects of human life with arts and humanities work on the interpretive, contextual, and critical dimensions of all these terms. The arts and humanities already have deep roots in this kind of study and as a group offer “a richer and more accurate account of happiness than is recognized in most of the current economic and psychological literature on the subject” (Small 2013, 176).

The next aspect of the happiness argument—the argument for enriched character development—is particularly tricky. Small points out that when character comes up in the debate between the sciences and humanities, the debate risks devolving into little more than character attacks. But it is legitimate to see that different forms of study create differently “trained habits of mind: distinctive intellectual priorities and tools to bear on distinctive kinds of object” (Small 2013, 58). As philosopher Martha Nussbaum (2010, xviii) puts it, “the humanities offer insights” and training of mind we “value as we seek to understand our lives.” They offer “a deeper understanding of love, death, anger, pain, and many other themes treated in great works of art, literature, and philosophy” (Nussbaum 2010, xviii).

We can add to this recent work in psychological humanities that points to the arts and humanities as invaluable tools for cultivating human personhood and practices of the self (Teo 2017; Sugarman and Martin 2020). The styles of personhood that can emerge from the arts and humanities include skill at interpretation and understanding, openness to difference and multiplicity of truths, tolerance for ambiguity, care for holistic understanding, and pleasure and curiosity in understanding individuals and unique moments in culture. In addition, Meretoja et al. (2022) have recently argued that literature, particularly meta-fiction, helps develop narrative agency skills in (1) narrative awareness, (2) narrative imagination, and (3) narrative dialogicality. These cultivations of personhood and trainings of mind can help us navigate the complexities of human life that other trainings of mind would not do.

The last of the arguments for happiness for the arts and humanities is that they can bring happiness and well-being directly. Small gives the example of John Stuart Mill and the way that the arts and humanities helped him in times of depression and despair. Mill found that in reading Wordsworth’s poems, “I felt myself at once better and happier as I came under [the poems’] influence …; [f]rom them I seemed to learn what would be the perennial sources of happiness” (Mill, quoted in Small 2013, 151). Small is careful to deflate claims that works of art are always a source of happiness and well-being or that these forms of happiness are somehow higher and superior to other forms of happiness. But this does not take away from the idea that, for many people, the arts and humanities do provide a kind of pleasure that other activities do not. For some, the arts and humanities are a high form of pleasure. This deflationary argument keeps the value of the arts and humanities for those who enjoy them without falling into superiority complexes. And the argument is consistent with the wealth of work being done in the arts-for-health movement, which is showing the many ways that the arts and humanities can be directly valuable for health and well-being (Clift and Camic 2016; Fancourt 2017; Arts and Humanities Research Council 2018; Billington 2016, 2019; Crawford, Brown, and Charise 2020).

Before leaving the happiness argument, it is important to add that the character development aspect of the arts and humanities is particularly critical for psychotherapy work. The styles of character and personhood that can emerge from the arts and humanities—such as skill at interpretation and understanding, openness to difference and multiplicity of truths, tolerance for ambiguity, care for holistic understanding, and pleasure and curiosity in understanding individuals and unique moments in culture—are all essential for psychotherapy work. Indeed, these character traits resonate with the common factors found in good therapists: empathy, understanding, collaboration, affirmation, emotional experience, and ability to form an alliance (Nahum, Alfonso, and Sonmez 2019; Oatley and Djikic 2018). This insight is compelling since the work of understanding another person in therapy overlaps with the work of understanding in the arts and humanities more broadly. There has been much work in health humanities and narrative medicine developing related insights into the value of the arts and humanities for training healthcare workers, but it seems even more directly related when training mental health workers. Certainly, it is easy to see that spending time with “A Nervous Breakdown” would enhance all of the common factors of good therapists mentioned above and therefore be an invaluable resource for psychotherapy training.

The fourth argument for the arts and humanities is the “democracy needs us” argument. The arts and humanities, from this perspective, help create democratic citizens who, as Nussbaum puts it, have “the ability to think critically; the ability to transcend local loyalties … and … the ability to imagine sympathetically the predicament of another person” (Nussbaum 2010, 7). Small is careful not to idealize this claim and points out the limits of this argument—its tendency toward the ideal of an elite guardian culture and its overinflated sense that the arts and humanities are the only way to develop these capacities. But Small does agree that “the humanities, centrally concerned as they are with the cultural practices of reflection, argument, criticism, and speculative testing of ideas, have a substantial contribution to make to the good working of democracy” (Small 2013, 7).

The argument for democracy particularly stands out in the context of recent mad-pride activist work and the emergence of critical mad studies scholarship. All of this critical work reveals ways in which mental health research, education, and practice are sources of deep political conflict and contention in contemporary culture (see Mad in America [n.d.] for examples of this political contention). What is important to understand is that recent mad pride activism and critical mad studies work are organized precisely around issues of democracy and sanist prejudice (Andersen et al. 2017; Beresford and Russo 2022; Russell, Ali, and Lewis forthcoming).

Recent mad studies work has moved beyond anti-psychiatry critiques organized around ontological claims, such as the “myth of mental illness,” to broader claims for democracy and epistemic justice. The question is less Is mental illness real or a myth? and more Who gets to decide? Who is included in the creation of mental health knowledge(s) and practices? Who is excluded? Why are these inclusions and exclusions created? How can we open our knowledge and practices surrounding mental differences to a greater diversity of perspectives? How can we include more disciplines, more methods of knowing, more points of view, and more approach alternatives? Most importantly, how can we include the perspectives of the key stakeholders—namely, those of us who are impacted by this knowledge and who are most unhappy and aggrieved with how such knowledge and practices have emerged? Bringing in the arts and humanities for our understanding of mental health improves these mad pride and mad studies democracy concerns by virtue of bringing in a wealth of new voices advocating for democracy. At their best, as Small points out, and as we saw with Chekhov’s “A Nervous Breakdown,” the arts and humanities can be powerful voices against prejudice and toward democratic inclusion.

The final argument Small articulates for the arts and humanities is that they are valuable “for their own sake” (see Small 2013, chapter 5). This argument speaks to the intrinsic worth of the arts and humanities, independent of their consequential values. This tautological or “just because” argument is complicated philosophically and readily breaks down if pushed very far but makes sense intuitively—the arts and humanities have worth and add value to the world just because the world without them (at least for many) is inherently less desirable. The arts and humanities are not the only way to create practices that feed into a good life—one thinks of sports or religious practice or gardening or appreciation of culinary delights as other options. But, and here is the tautology, for those whom the arts and humanities help create a good life, the arts and humanities are part of a good life. For us, as authors, spending time with Chekhov’s “A Nervous Breakdown” is part of a good life. Life without Chekhov’s stories and plays, independent of any use value that they might have, would not be as good. Not everyone would feel this way, but some people do. For those people, the arts are part of a good life.

## Conclusion

It seemed, not so long ago, that empirical natural science and quantitative research were the only way forward for understanding mental health and that neuroscience would lead the way. But now, with a wealth of research on the limits of neuroscience for understanding mental health, it is time to reconsider how we research, teach, and practice mental health care.

Allowing ourselves to learn from Chekhov’s work, we can see how *mental health humanities* emerges as a way forward. Chekhov’s grounding in both literature and medical science opens the opportunity of embracing the complexity and multiplicity of approaches. From this perspective, cognitive science, behavioral science, neuroscience, genetics, and medical science all have their place, and they all do what they can to help explain and predict aspects of mental difference. At the same time, the arts and humanities also provide us with a valuable understanding that cannot be achieved in any other way. This hardly means that any work in the arts and humanities should be embraced uncritically, but it does mean that, as we can learn about mental difference, mental health, and mental healthcare from the sciences, we can also learn from the arts and humanities.

Now is the time to move forward in this direction through creating communities, similar to what has happened in the health humanities and disability studies. Although their focus is physical health and physical difference, these domains have also started giving attention to mental health and mental difference. The significance of mental health for the health humanities can be seen in projects like Creative Practice for Mutual Recovery, the Madness & Literature Network, and the Dementia Arts & Wellbeing Network, showcased by the International Health Humanities Network (n.d.). Similarly, using the signifier *mad humanities*, Hayley Stefan brings together disability studies with mad studies to articulate the role that “mad humanities” and “mad literary studies” can play in developing these connections (Stefan 2018). Important steps are also being made in psychology through the development of *psychological humanities* and recent works devoted to reading and mental health (Teo 2017; Healy 2017; Sugarman and Martin 2020; Billington 2016, 2019).

By setting up related communities around the world that include a broad base in the arts and humanities as well as mad pride activists and scholars, we can make meaningful steps to expand the research, education, and practice of mental health. If Vasilyev were to live in a future world that included mental health humanities, he would have met mental health workers exposed to more than natural-science approaches. Vasilyev would have a greater possibility of being understood, being connected to others with related concerns, and avoiding the kind of scientist dogmatism he met in the story’s psychiatrist.

## Data Availability

Not applicable.
